# Metabolite Comparison between Serum and Follicular Fluid of Dairy Cows with Inactive Ovaries Postpartum

**DOI:** 10.3390/ani12030285

**Published:** 2022-01-24

**Authors:** Zhijie Wang, Yuxi Song, Shuhan Sun, Chang Zhao, Shixin Fu, Cheng Xia, Yunlong Bai

**Affiliations:** 1 College of Animal Science and Veterinary Medicine, Heilongjiang Bayi Agricultural University, Daqing 163319, China; wangzhijie97@163.com (Z.W.); syxalz@163.com (Y.S.); easonren9807@163.com (S.S.); fushixin@163.com (S.F.); 2 College of Animal Science and Technology, Anhui Agricultural University, Hefei 230036, China; chang_zhao@ahau.edu.cn; 3 Heilongjiang Provincial Key Laboratory of Prevention and Control of Bovine Diseases, Heilongjiang Province Cultivating Collaborative Innovation Center for The Beidahuang Modern Agricultural Industry Technology, Daqing 163319, China

**Keywords:** dairy cows, inactive ovaries, serum, follicular fluid, metabolites

## Abstract

**Simple Summary:**

Although the milk production of dairy cows has increased rapidly in recent decades, the reproductive performance of dairy cows has gradually declined. In modern intensive dairy farms, prevention and treatment of inactive ovaries has become an important challenge of reproduction disorders during early lactation. Our aim is to screen out metabolites and metabolic pathways related to inactive ovaries through serum and follicular fluid metabolomics. We found that the changes in serum and follicular fluid were mainly enriched in nine metabolic pathways. In serum, these included d-glutamine and d-glutamate metabolism, alanine, aspartic and glutamate metabolism, arginine and proline metabolism, pentose and glucuronate interconversions, and glycerophospholipid metabolism. In follicular fluid, they were valine, leucine, and isoleucine biosynthesis; arachidonic acid metabolism; glycerophospholipid metabolism; starch and sucrose metabolism; phenylalanine metabolism; and pentose and glucuronate interconversion. The common metabolic pathways of disease-related serum and follicular fluid were pentose and glucuronate interconversions and glycerophospholipid metabolism. This research will provide a theoretical basis for exploring the causes of inactive ovaries and provide new ideas for the prevention and treatment of inactive ovaries in the future.

**Abstract:**

Inactive ovaries (IO) accounts for 50% of ovarian disease in postpartum dairy cows, which seriously affects their reproductive efficiency. To investigate the metabolic changes in the serum and follicular fluid of dairy cows with IO during lactation, six estrus (E) cows and six IO cows at 50 to 55 days in milk were selected based on B ultrasonic detection and clinical manifestations. The differential metabolites in serum and follicular fluid between the E cows and IO cows were identified by ultra-high-pressure liquid chromatography–quadrupole time-of-flight mass spectrometry, combined with multidimensional statistical methods. The results showed that dairy cows with IO were in a subclinical ketosis status where beta-hydroxybutyrate (BHB) exceeded 1.20 mmol/L, 14 differential metabolites in the serum of IO cows included 10 increased metabolites and 4 decreased metabolites, and 14 differential metabolites in the follicular fluid of IO cows included 8 increased metabolites and 6 decreased metabolites. These differential metabolites mainly involved nine metabolic pathways. The common enrichment pathway of different metabolites in serum and follicular fluid were glycerophospholipid metabolism and pentose and glucuronate interconversions. In conclusion, there were significant differences in the differential metabolites and enrichment pathways between serum and follicular fluid of IO cows, implying that there were complex changes in blood metabolism and local follicular metabolism of IO cows, whose interactions need further investigation.

## 1. Introduction

With the rapid increase of milk production in dairy cows, the reproductive performance has gradually declined in the past few decades [1]. Cows with high milk production usually have low postpartum reproductive efficiency, which seriously impacts the economic development of the dairy industry [1]. Preovulatory reproductive failure due to cow metabolic changes in the transition period potentially affects the timing of the return to estrus [2]. It is known that inactive ovary (IO) is a postpartum ovarian disease in dairy cows, which sometimes accounts for 50% of ovarian disease [3]. For 50 to 55 days postpartum, cows do not show estrus and there is no corpus luteum on the surface of the ovaries. Follicle waves appear on the surface of the ovaries, but the growth of the follicles stops before the follicles deviate, termed IO [4], so on modern intensive dairy farms, prevention and treatment of ovarian inactivity has become an important challenge of reproductive disorders during early lactation.

Follicular fluid (FF) is the microenvironment for the development and maturation of oocytes in animals and humans. It contains biologically active molecules and proteins that may affect follicular growth and oocyte fertilization and can affect the quality of oocytes, fertilization, and possibly even the development of embryos [5]. As the follicle develops, the composition of the FF will change [6,7], proving that it participates in the development of the follicle and oocyte and is therefore closely related to oocyte meiosis, ovulation, corpus luteum formation, and fertilization [8]. The metabolites contained in FF mainly come from blood and secretions by theca cells, granulosa cells (GCs), and the oocyte and include lipids, glucose, proteins, cytokines, steroids, growth factors, and peptide hormones [9,10,11,12], so it is necessary to jointly analyze the similarities and differences between the metabolism of follicular fluid and serum of dairy cows with IO during early lactation.

Metabolomics technology has developed into a mature technology in the past decade that can directly detect the physiological and pathological states of individuals, provide the most comprehensive and direct characterization, and provide information about the pathological changes and mechanisms of disease occurrence [13]. Luo et al. [14] reported on plasma metabolite changes in cows during parturition, providing new information on pathways for the initiation of physiological responses under the stress of parturition. Ametaj et al. [15] used metabolomic methods to reveal changes in plasma amino acid and sphingolipid profiles in transitional diseased cows, providing evidence for selected amino acids as biomarkers of disease or deviation from normal health status.

Currently there have been no reports on metabolic changes between in blood and in FF simultaneously of ovarian diseases in dairy cows during early lactation, so this study aimed to expose metabolic changes during IO using liquid chromatography–mass spectrometry (LC/MS) technology and to compare differential metabolites (DMs) of serum and FF of postpartum IO cows by statistical analysis and information biology analysis.

## 2. Materials and Methods

### 2.1. Sample Collection and Clinical Information

This experiment was carried out using Holstein cows on a large intensive cattle farm in the central region of Heilongjiang Province according to the requirements of the Veterinary Medical Ethics Committee of the Ministry of Agriculture of China. All experiments on animals were conducted according to the standards approved by the Animal Welfare and Research Ethics Committee at Heilongjiang Bayi Agricultural University (No. 20200127). The total mixed ration (TMR) of tested dairy cows complied with the NRC (2001) [16] and with the Chinese feeding standard for dairy cows. The composition and chemical components of the TMR diet included 1.03 kg cottonseed, 1.50 kg soybean husk, 2.50 kg alfalfa, 1.30 kg soybean meal, 2.00 kg corn flakes, 1.00 kg molasses, 25.37 kg silage, and 3.00 kg corn, with a milk net energy of 7.322 MJ/kg.

The body condition score (BCS) was measured on a five-point scale of one (thin) to five (obese), with intervals of 0.25 used to assess body fat stores [17]. At 50 to 55 d postpartum as the time for the first estrus and breeding of the cow after uterine involution, the milk yield was recorded on the same day as blood sample collection for serum hormone detection. Screening for persistent corpus luteum, ovarian atrophy, corpus luteum cysts and follicular cysts, and uterine-related diseases was conducted by B-ultrasonic detection and rectal examination. Cows with abnormal serum biochemical indexes were excluded through serum energy indexes, trace element indexes, liver function indexes, etc.

Six healthy cows with estrus behaviors and no signs of disease or clinical abnormalities were selected as the estrus (E) group, based on the fluctuations in the step count at 50 to 55 d postpartum and clinical manifestations of estrus, such as crawling or moist and swollen mucous membranes of the labia. B-ultrasonography examination was used to examine the boundaries between the muscularis and intima layers of the cervix, thickened uterine wall, uneven hardness, fluid in the uterine cavity, and ovarian follicles with a diameter of 15 to 20 mm in E cows. Six cows with IO were selected as the IO group, on the basis of no significant fluctuation at 50 to 55 d postpartum, no clinical estrus by the Afimilk Ranch Management System (Afimilk^®^3.076, Afimilk, Israel), no fluid in the uterine cavity, normal ovarian hardness and size, follicle diameter less than 8 mm, and follicle enlargement less than 3 mm within five days by B-ultrasound examination of the endometrial folding [18]. The daily follicular growth rate was calculated as the difference between the largest follicle diameters on d 50 and d 55 divided by five.

The blood samples were collected with a syringe through the coccygeal vein of the cows in the morning before feeding and centrifuged at 3000 rpm for 10 min to collect the serum into 1.5 mL tubes, then recentrifuged at 12,000 rpm for 10 min to collect supernatant to store at −80 °C. The follicular fluid of the IO cows was collected in the secondary follicle stage [18]. At 50 to 55 d postpartum, a transvaginal ultrasound-guided aspiration technique (Sonosite-Titan, 10 mHz, micro convex probe, Bothell, WA, USA) was used on the two groups of cows to collect FF after measuring the follicular diameter and centrifuged at 12,000 rpm for 15 min to collect the supernatant to store at −80 °C for proteomic analysis [19,20]. Clinical information such as milk yield, body condition, age, and diseases was collected at the same time by the Afimilk Ranch Management System.

The test data were statistically analyzed using IBM SPSS 23.0 (IBM, Armonk, NY, USA). The independent sample *t*-test was used to analyze the significance of differences in clinical background information, including age, parity, body condition score, milk yield, follicular growth status, and serum indexes between the E and IO groups, and the data were expressed as mean ± standard error.

### 2.2. Blood Biochemical Index Detection

The serum biochemical indicators measured included those involved in energy metabolism, including β-hydroxybutyrate (BHB), non-esterified fatty acid (NEFA), and glucose (Glu); trace elements, including calcium, phosphorus, and magnesium; liver function, including alanine aminotransferase (ALT), aspartate aminotransferase (AST), and total protein (TP); and reproductive hormones, including estradiol and progesterone. Serum BHB was measured using a serum ketone meter and ketosis reagent strips with 93.8% sensitivity, 100% specificity, and a 93.8% Youden index (Yicheng, Beijing, China). Serum NEFA, Glu, calcium, phosphorus, magnesium, ALT, AST, and TP were measured using commercial kits from Biosino Biotechnology and Science Inc., Beijing, China.

### 2.3. Sample Pre-Treatment

At first, 100 μL of either serum or FF was pipetted into a centrifuge tube and 300 μL of methanol mixture containing the internal standard l-2-chlorophenylalanine 1 μg/mL was added, vortexed, and mixed for 30 s. Then, the mixture was then placed in an ice-water bath and sonicated (Shenzhen Redbond Co. Ltd., PS-60AL, Shenzhen, China) for 10 min and then incubated for one hour in a refrigerator at −40 °C. Next, the test sample was centrifuged at 12,000 rpm for 15 min at 4 °C and finally, 60 μL of the supernatant was drawn into a sample bottle for testing on a Q Exactive HFX mass spectrometer (AB Sclex, Redwood City, CA, USA). Finally, a quality control sample (QC) was created by taking an equal amount of supernatant from all samples and mixing them together for mass spectrometer detecting.

### 2.4. Detection of Serum and FF by LC/MS

Ultra-high-pressure liquid chromatography (Vanquish, Thermo Fisher Scientific, Waltham, MA, USA) with a liquid chromatograph 2.1 mm × 100 mm, 1.7 μm column (Waters ACQUITY UPLC BEH Amide, Milford, MA, USA), were used to separate the serum and FF samples from the test cows. The Q Exactive HFX mass spectrometer (AB Sclex, Redwood City, CA, USA) performed primary and secondary mass spectrometry data acquisition using the control software (Thermo, Xcalibur, Waltham, MA, USA). In NCE mode, the voltage was 3.5 kV in positive ion mode and −3.2 kV in negative ion mode. For detailed experimental steps, refer to the previous study by Bai et al. from 2020 [21].

### 2.5. Differential Screening and Statistical Analysis of Data

The SPSS 23.0 software (IBM, Armonk, NY, USA) was used for statistical analysis of clinical information and serum biochemical indicators collected from experimental cows via one-way ANOVA. The data are shown as the mean ± standard deviation. After obtaining the finished metabolomics data, multivariate statistical analysis was performed. The SIMCA software (V15.0.2) was used to perform logarithmic (LOG) conversion and centralization (CTR) format processing on the data and automatic modeling and analysis. Principal component analysis (PCA) and orthogonal partial least squares–discriminant analysis (OPLS-DA) were used for the results. Seven-fold cross validation was used to test the model, cross-validation Q^2^ and R^2^Y were used to determine whether the model was valid, and then through a permutation test, the order of the categorical variable Y was randomly changed several times to obtain the R and Q values of the random mode. The false discovery rate (FDR) was applied to correct the results. Finally, a *t*-test was performed on the metabolomics data to further check that the model was effective. The final selection criteria for DMs were that *p* < 0.05 and the variable projection importance (VIP) of the OPLS-DA model was greater than one.

### 2.6. Correlation Analysis between DMs and Metabolic Pathway Analysis

The compounds were screened by the Human Metabolome Database (HMDB) and the Madison Metabolomics Consortium Database (MMCD). MetaboAnalyst 3.0 was used to analyze the DMs selected by multivariate statistical analysis and univariate analysis. Visual analysis of metabolites was carried out using hierarchical clustering and a heatmap. Through the Kyoto Encyclopedia of Genes and Genomes (KEGG) pathway database, all pathways were selected corresponding to the mapping of DMs and conduct topological analysis and enrichment analysis on the pathways of the DMs. The metabolic pathways were further screened to find the key metabolic pathways with the highest correlation with metabolites.

## 3. Results

### 3.1. Clinical Information

The clinical parameters, including age, parity, BCS, and milk yield, are shown in Table 1. The differences among the two groups in terms of number of dairy cows, age, parity, and BCS were not significant (*p* > 0.05), but cows with IO had a higher daily milk yield (*p* < 0.05).

### 3.2. Follicle Development Information

Table 2 shows the follicular development information of 12 dairy cows 50 to 55 d postpartum. The diameter of the dominant follicles in the IO group was significantly smaller than in the E group (*p* < 0.01). The growth rate of dairy cows in the IO group was significantly slower than in the E group (*p* < 0.01).

### 3.3. Serum Biochemical Indicator Levels

Table 3 shows the serum biochemical indexes of the two groups of cows. The serum glucose concentration of the IO group was lower than that of the E group (*p* < 0.05), BHB and NEFA concentrations were higher than those of the E group (*p* < 0.05), and there was no difference in other indicators between the two groups.

### 3.4. Analysis of Serum and FF Metabolomics

#### 3.4.1. Orthogonal Partial Least Squares Discriminant Analysis and Permutation Test

**Figure 1:**
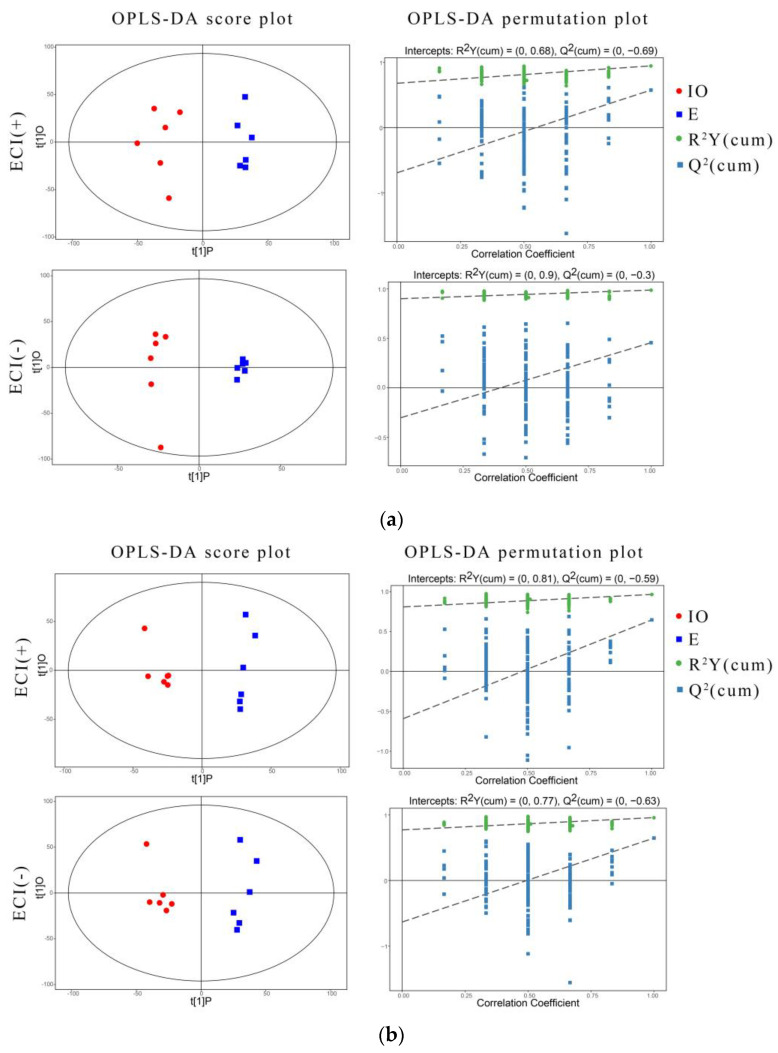
OPLS-DA model analysis of the E group versus the IO group in positive and negative ion mode in serum (**a**) and follicular fluid (**b**) samples. The prediction score of the first principal component of the scatter plot is the abscissa, the orthogonal principal component score is the ordinate, red is the E group, and blue is the IO group. In the OPLS-DA permutation plot, the permutation retention rate is the abscissa, the value of R^2^Y or Q^2^ is the ordinate, the blue dot is the Q^2^ value, the green dot is the R^2^Y value, and the dotted line is the regression line.

The scatter plots and permutation tests of OPLS-DA models in the E group and the IO group are shown in Figure 1. The results of the OPLS-DA score chart show that the two groups of serum and FF samples in the positive and negative ion modes were clearly distinguished and both were in the confidence interval of 95%. The R^2^Y of the serum positive ion mode, negative ion mode, FF positive ion mode, and negative ion mode were 0.68, 0.9, 0.81, and 0.77, respectively, which were all close to one. This showed that the constructed models were in line with the true sample data and the intercept of the Q^2^ regression line and the vertical axis of the positive and negative ion model were less than zero. When the replacement retention decreased, the proportion of the Y variable gradually increased and Q^2^ gradually decreased, which shows that the built model had good robustness and no over-fitting phenomenon.

#### 3.4.2. DM Screening

Table 4 shows the different metabolites in the serum and FF of the two groups of experimental cows. A total of 28 different serum and FF metabolites were screened in the two models, of which 18 were increased and 10 were decreased. The serum of the cows in the IO group had increased levels of l-glutamine (*p* = 0.0222), guanidinoacetic acid (*p* = 0.0153), citrulline (*p* = 0.0060), l-arginine (*p* = 0.0411), l-glutamic acid (*p* = 0.0216), alpha-ketoisovaleric acid (*p* = 0.0299), (R)-3-hydroxybutyric acid (*p* = 0.0074), lactosylceramide (d18:1/16:0) (*p* = 0.0178), phosphatidylcholine (*p* = 0.0112), and sphingomyelin (d18:1/18:1(9Z)) (*p* = 0.0387) and decreased levels of 4-pyruvate (*p* = 0.0492), indoleacetic acid (*p* = 0.0404), d-xylose (*p* = 0.0252), and l-ribulose (*p* = 0.0085). The FF of the cows in the IO group had increased levels of l-glutamine (*p* = 0.0337), choline (*p* = 0.0100), lysophosphatidylcholine (14:1(9Z)) (*p* = 0.0069), phosphatidylcholine (22:4(7Z,10Z,13Z,16Z)/14:0) (*p* = 0.0403), phosphatidyl ethanolamine (18:4(6Z,9Z,12Z,15Z)/P-18:1(11Z)) (*p* = 0.0153), arachidonic acid (*p* = 0.0435), d-maltose (*p* = 0.0276), and urocanic acid (*p* = 0.0202) and decreased levels of l-valine (*p* = 0.0001), ketoleucine (*p* = 0.0018), phenylpyruvic acid (*p* = 0.0330), gentisic acid (*p* = 0.0233), 11,12-DiHETrE (*p* = 0.0098), and 6-hydroxy-5-methoxyindole glucuronide (*p* = 0.0043).

#### 3.4.3. Pathway Analysis

**Figure 2:**
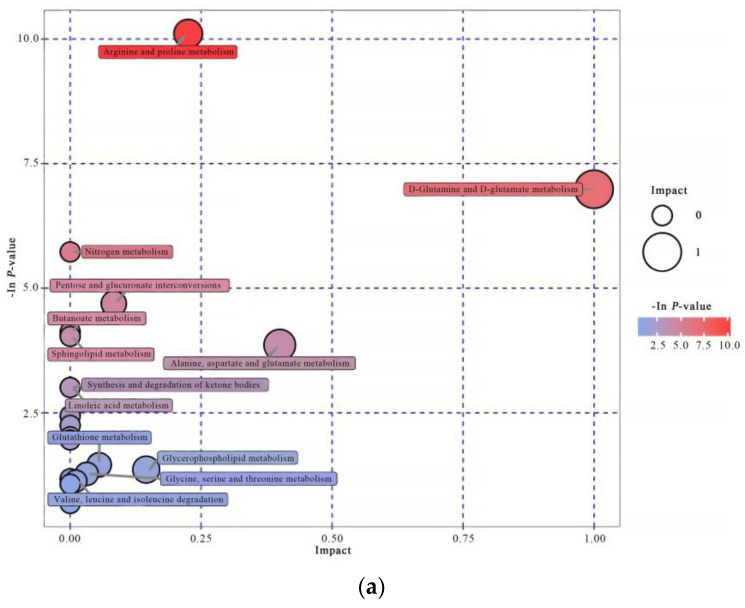

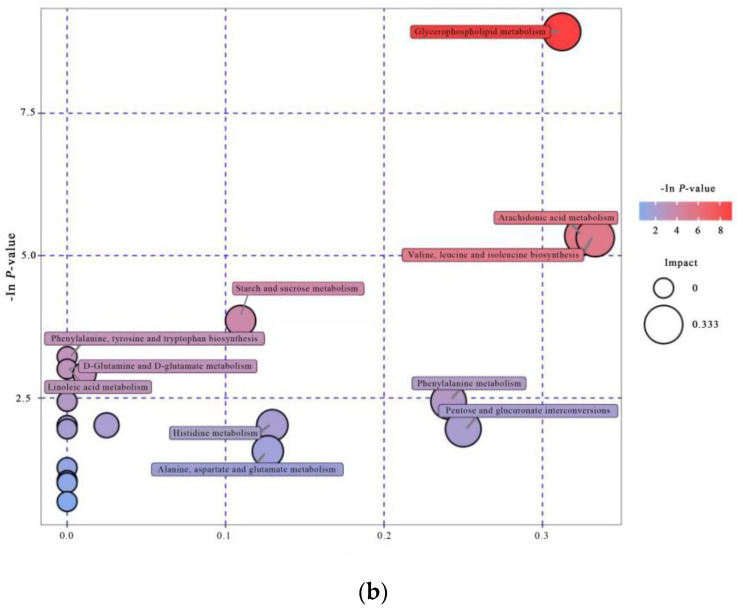
Bubble diagrams of serum (**a**) and follicular fluid (**b**) samples. The position and size of the bubble on the abscissa is the size of the influence factor of the path in the topological analysis. The larger the bubble, the greater the influence factor. The position and color of the bubble on the ordinate is the *p*-value in the enrichment analysis (-ln *p*-value, i.e., negative natural logarithm); the smaller the *p*-value, the darker the color, and the more significant the enrichment.

Using KEGG pathway analysis, the different metabolites in the pathway between IO cows and E cows were identified and represented by bubble diagram. The pathway analysis is shown in Figure 2. In serum, these included d-glutamine and d-glutamate metabolism, alanine, aspartic and glutamate metabolism, arginine and proline metabolism, pentose and glucuronate interconversions, and glycerophospholipid metabolism. In FF, they included valine, leucine and isoleucine biosynthesis, arachidonic acid metabolism, glycerophospholipid metabolism, starch and sucrose metabolism, phenylalanine metabolism, and pentose and glucuronate interconversion. The common metabolic pathways of disease-related serum and FF were pentose and glucuronate interconversions and glycerophospholipid metabolism.

## 4. Discussion

Follicle-stimulating hormone increases after cows’ parturition, lasting two to three days. When the follicle grows to a certain stage, the difference between the growth rate of the largest follicle and the second largest follicle reaches the maximum and follicle deviation occurs [22]. The maximum diameter of the follicle may decrease due to the decrease in luteinizing hormone (LH) pulse and the dominant follicles may fail to ovulate due to inadequate LH production. Hormone secretion disorder is only one of the direct causes of ovulation failure, but there are still many other factors that affect the development of follicles.

High-producing cows with subclinical ketosis have low cholesterol or high liver perfusion, which may reduce steroid hormones synthesis or increase the clearance rate of ovarian steroid hormones and then lead to anovulation and persistent corpus luteum [23]. High-yield lactating cows cannot provide sufficient energy for follicle development and hinder the growth of follicles [24], which is consistent with these results. By comparing the serum biochemical indicators of E cows and IO cows in this study, the serum BHB and NEFA levels of IO cows were shown to be higher, whereas the glucose was lower, suggesting an obvious subclinical ketosis (SCK) feature [25,26]. Studies by Chang et al. [27] also confirmed that cows developed SCK at 14 to 21 days postpartum and 50% of them showed IO during postpartum at 60 to 90 days. Serum BHB and NEFA can inhibit the survival and growth of sheep preantral follicles and their oocytes cultured in vitro [28]. Glucose, total cholesterol, and BHB affect oocyte maturation ability in dairy cows [29].

On the other hand, we observed higher milk production in IO cows, which may also be one of the main factors affecting follicular development. Forde et al. [30] found that lactation induces distinct changes in the overall metabolic status of postpartum dairy cows. These changes create a different microenvironment for the developing oocyte and presumably contribute to lower fertility in some cases.

### 4.1. Changes in Serum Metabolites in IO Cows

Amino acids are one of the main components of protein and milk fat. In early reports, some amino acids were shown to affect the milk quality and production performance of dairy cows [29,31]. In this study, there were seven serum DMs directly involved in amino acid metabolism in IO cows. Previous studies have confirmed that glutamine and glutamate can be converted into each other and directly participate in the tricarboxylic cycle for gluconeogenesis [32]. Guanidinoacetic acid can generate creatine in the form of phosphocreatine and free creatine, which provide energy for the body, and alpha-ketoisovaleric acid can be converted into valine, and together with isoleucine, it can regulate the blood-sugar level to provide energy for the body [33,34]. This study found that the energy metabolism of IO cows was disturbed, and the urea cycle was also affected, by affecting the formation of fumaric acid through amino acids such as arginine and participating in the tricarboxylic cycle [35], but also by being involved in the synthesis of antioxidants and anti-inflammatory factors [36,37]. The specific mechanism by which amino acid metabolism affects inactive ovaries through energy metabolism or inflammation is unclear and requires further investigation.

The carbohydrates screened in this experiment mainly involve pentose and glucuronate interconversions. The pentose phosphate pathway can offer raw materials for the synthesis of other substances, such as nucleotides and amino acids for energy provision [38]. In addition, l-ribulose can generate ribitol and carry out riboflavin metabolism, which participates in the energy response of the respiratory chain and cell growth and metabolism [39]. The blood l-ribulose and d-xylose levels are reduced to maintain the level of pyruvate, and some metabolic pathways such as the pentose phosphate pathway and riboflavin metabolism are weakened, which is not conducive to follicular development.

The study by Luo et al. [14] showed that the content of phosphatidyl-choline in the serum of dairy cows increased during early lactation, which is consistent with our results. Phosphatidylcholine homeostasis is particularly important for maintaining cell survival and growth, and studies have found that the total amount of phosphatidylcholine in cells is positively related to cell growth and negatively related to apoptosis [40]. Under the action of phospholipase, phosphatidylcholine can increase the production of arachidonic acid and linoleic acid [41]. The 13(S)-hydroperoxyoctadecadienoic acid produced by the oxidative metabolism of linoleic acid can enhance epidermal growth factor signal transduction by participating in the dephosphorylation of epidermal growth factor receptors and inducing the expansion of the cumulus in the ovaries of mammals [42]. Sphingomyelin can be metabolized to produce ceramide, which has important functions in barrier function, regulating cell function and participating in the signal transduction process, which regulates cell growth and apoptosis [43]. When culturing cells in vitro, adding ceramide can cause protein kinase inactivation and reduce the absorption of glucose by cells and accelerate cell apoptosis [43], but the relationship between ceramide and IO in dairy cows needs further study. This study found that 4-pyridoxine, a metabolite of vitamin B6 formed from pyridoxal by aldehyde oxidase (AOX) in the liver, decreased in the serum of cows with IO. Its relationship with IO needs to be further explored.

### 4.2. Changes in FF Metabolites in IO Cows

In this study, the change trend of glutamine in FF and serum was consistent. l-glutamine is the precursor and main energy source of nucleic acid biosynthesis, which enters the glycolysis and gluconeogenesis pathways or the purine and pyrimidine metabolism pathway. It can provide energy for the body and promote the proliferation of cumulus cells in vitro [44,45]. Excessive glutamine is transported to the cell through the transport system alanine–serine–cysteine (ASC), which may act as a competitive inhibitor of cysteine uptake. Cysteine is a key factor in the synthesis of glutathione in the g-glutamyl cycle [46], but it is still unclear whether glutamine in dairy cows with IO has an effect on the synthesis of glutathione, which in turn oxidizes and damages the follicular cells and hinders the development of the follicle. Other metabolites, such as l-valine, phenylpyruvic acid, and gentisic acid, can all participate in the tricarboxylic cycle, which in turn affects carbohydrate metabolism and lipid metabolism [47,48].

The carbohydrates present in FF are mainly related to starch and sucrose metabolism, pentose and glucuronate interconversions, and d-maltose as an intermediate substance that can convert glycogen into glucose. During the maturation of oocytes, both glucose and 6-hydroxy-5-methoxyindole glucuronide can provide energy for cells. Glucose can also synthesize extracellular matrix substrates by cumulus expansion and O-linked glycosylation in cell-signal transduction through the hexosamine biosynthesis pathway for follicular growth and regulate oocyte nuclear maturation and redox state through the pentose phosphate pathway, and 6-hydroxy-5-methoxyindole glucuronide generates ascorbic acid, which resists oxidation and scavenges free radicals [49,50]. In this study, the increase in maltose in the FF of IO cows and the decrease in 6-hydroxy-5-methoxyindole glucuronide may have enhanced glycogenolysis.

Lipid-related metabolites are mainly involved in glycerophospholipid and arachidonic acid metabolism. Phosphatidylinositol can be produced from serine and then enter the tricarboxylic cycle through acetyl-CoA or indirectly produce choline. It can also participate in the glycosylphosphatidylinositol (GPI) metabolic pathway, allowing cell membranes to bind to proteins [51,52]. Studies have shown that choline can generate acetylcholine and enter the cAMP signaling pathway. Acetylcholine is a neurotransmitter that not only affects the permeability of the membrane to ions, but also transmits signals through some second messengers and affects the physiological metabolic process. Studies have shown that in the ovaries cAMP is instrumental as a second messenger for the follicle-stimulating hormone (FSH) and luteinizing hormone (LH) receptors [53,54,55]. The increased metabolism of glycerophospholipids may be related to signal transduction during follicular development of IO cows with SCK, but further research is needed. Arachidonic acid is elevated, which is consistent with the results of Moore et al. [20]. Arachidonic acid participates in the ovarian production of steroid hormones through its metabolites, such as cyclooxygenase metabolism, to produce PGE_2_ and PGF_2α_ and lipoxygenase metabolism to produce leukotrienes. PGE_2_ can promote the synthesis of hyaluronic acid and the expansion of the cumulus or 5-hydroperoxyeicosatetraenoic acid produced by metabolism, which can affect the production of steroid hormones by regulating the expression of steroidogenic acute regulatory protein [56,57]. However, Zhang et al. [58] pointed out that high concentrations of arachidonic acid can induce the death of granulosa cells in the ovary and inhibit the synthesis of estrogen by granulosa cells. This result may be because the catabolism of arachidonic acid is decreased and the content of FF is increased, which affects the synthesis of steroid hormones in granular cells. Arachidonic acid is the precursor of many circulating eicosanoids derivatives. A review of FF by Guerreiro et al. [59] showed that the inflammatory status of the animals does indeed play a key role in oocyte production and, ultimately, survival. Docosanoids and eicosanoids, the molecular classes observed in the low-oocyte group, are well-known players involved in reactions that lead to an inflammatory state in organisms.

### 4.3. Comparison of Metabolites in Serum and FF in IO Cows

**Figure 3:**
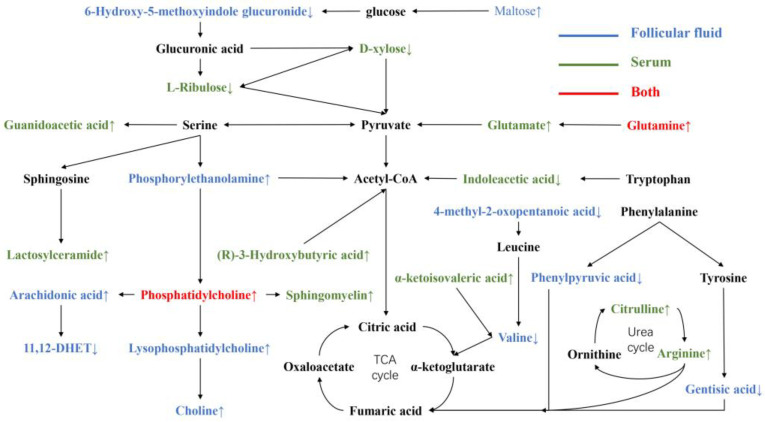
Differential metabolite network metabolism map in dairy cows with inactive ovaries. ↑ and ↓ represent increase and decrease, respectively. Blue means follicular fluid samples, green means serum samples, and red means both. Black means no difference between serum and follicular fluid.

In this experiment, UHPLC-QTOF-MS was used to analyze the serum and FF samples of the E and IO cows, and 28 different metabolites were obtained. Using KEGG analysis of metabolic pathways, an interaction correlation diagram between DMs was constructed through integration, as shown in Figure 3. Glutamate and phosphorylcholine were the only common DMs in serum and FF in this study, which is consistent with the findings of Bender et al. [60], in which the comparison of serum and FF fatty acid profiles showed that the composition of follicular fluid is unique and that it is not a simple reflection of differences in serum profiles. Because the metabolites of FF come partly from serum and partly from local cells, this means that the metabolic activity of the follicular cells has an impact on its composition.

Scaramuzzi et al. [61] pointed out that the SCK affects the growth of follicles induced at different levels of the hypothalamic–pituitary–ovarian axis. Rodgers and Irving-Rodgers [62] reviewed the regulation of follicular growth, fertility, and oocyte quality in ruminants. The FF from the blood needs to pass through the cortical interstitium, the basal layer of the follicle, and the granular cell layer of the wall, and as the follicle develops [63], fluid will accumulate in the antral cavity of the follicle to provide nutrients for the development and maturation of the oocyte. The main function of follicles is to provide a blood–follicle barrier and create a favorable environment for growing oocytes. Similarly, oocytes play an important role in promoting the growth of follicles and directing the differentiation of granulosa cells and interact with surrounding somatic cells such as granulosa cells [63]. In this study, l-glutamine and l-glutamate in the FF and serum were increased in dairy cows with IO, which was not conducive to follicular development. The increased l-glutamine in FF should derive from l-glutamate in the blood and then be converted to pyruvate, which can provide energy for follicular development [32], mainly via alanine, aspartate, and glutamate metabolism. In IO cows, the serum and FF phosphatidylcholine and its upstream and downstream metabolites were all elevated, mainly due to arachidonic acid metabolism. Although an appropriate concentration of arachidonic acid can participate in the production of steroid hormones, the high content of arachidonic acid in FF can induce granular cell death, which is not conducive to follicular development [56,57].

## 5. Conclusions

The main pathways involved were d-glutamine and d-glutamate metabolism, alanine, aspartic and glutamate metabolism, arginine and proline metabolism, valine, leucine and isoleucine biosynthesis, phenylalanine metabolism, glycerophospholipid metabolism, arachidonic acid metabolism, pentose and glucuronate interconversions, and starch and sucrose metabolism. Among them, glycerophospholipid metabolism mainly involved signal transduction during follicular development. This study found that sphingomyelin and lactosylceramide may be related to IO in dairy cows, but it is still unclear how the specific DMs affect IO development. Further in-depth study that expands the number of cows, as well as other research, is necessary in the future. The different enrichment pathways of serum and FF metabolic difference implies that there are the complex interactions between the blood metabolism and local follicular metabolism of IO cows, which needs to be further investigated.

## Figures and Tables

**Table 1:** Clinical information in the two groups of tested cows.

Project	Estrus Cows (*n* = 6)	Inactive Ovary Cows (*n* = 6)
Age (years)	3.37 ± 0.54	3.13 ± 0.92
Parity	2.33 ± 0.52	2.00 ± 0.89
BCS ^1^	2.92 ± 0.49	2.67 ± 0.20
Milk yield (kg/d)	38.48 ± 3.47	44.00 ± 4.17 *

^1^ Body condition score. * *p* < 0.05.

**Table 2:** Follicular development at 50 to 55 d postpartum.

Follicular Diameter	Estrus Cows (*n* = 6)	Inactive Ovary Cows (*n* = 6)
50 d postpartum (mm)	6.67 ± 1.70	5.33 ± 0.56
55 d postpartum (mm)	13.67 ± 0.71	7.33 ± 0.42 **
Growth rate (mm/d)	1.40 ± 0.21	0.40 ± 0.17 **

** *p* < 0.01.

**Table 3:** Serum biochemical parameter levels in the two groups of tested cows.

Project	Estrus Cows (*n* = 6)	Inactive Ovary Cows (*n* = 6)
BHB (mmol/L)	0.78 ± 0.37	1.37 ± 0.49 *
NEFA (mmol/L)	0.44 ± 0.09	0.73 ± 0.25 *
Glucose (mmol/L)	3.63 ± 0.35	2.99 ± 0.20 *
Calcium (mmol/L)	2.07 ± 0.28	2.12 ± 0.23
Phosphorus (mmol/L)	1.85 ± 0.32	1.63 ± 0.22
Magnesium (mmol/L)	1.22 ± 0.11	1.20 ± 0.09
ALT (U/L)	16.83 ± 7.78	10.31 ± 1.21
AST (U/L)	52.67 ± 21.59	41.00 ± 9.70
TP (g/L)	55.05 ± 17.71	44.07 ± 9.41

* *p* < 0.05. BHB = β-hydroxybutyric acid; NEFA = non-esterified fatty acids; ALT = alanine aminotransferase; AST = aspartate aminotransferase; TP = total protein.

**Table 4:** Differential metabolites in the serum and FF of the IO and E groups.

Category	ID	Metabolites	FC	*p*-Value ^b^	RT (min)	VIP ^a^	FD ^c^	Mode
Serum	1	l-glutamine	0.534	0.022	393.81	1.48	↑	ESI+
	2	Guanidoacetic acid	0.606	0.015	367.36	1.31	↑	ESI+
	3	Citrulline	0.470	0.006	429.12	1.63	↑	ESI+
	4	l-arginine	0.674	0.041	548.22	1.40	↑	ESI+
	5	l-glutamic acid	0.412	0.002	393.74	1.81	↑	ESI+
	6	4-Pyridoxic acid	1.493	0.049	34.89	1.73	↓	ESI−
	7	Indoleacetic acid	5.658	0.040	149.56	2.47	↓	ESI−
	8	Alpha-ketoisovaleric acid	0.460	0.030	64.89	1.83	↑	ESI−
	9	(R)-3-hydroxybutyric acid	0.611	0.007	247.68	2.10	↑	ESI−
	10	Lactosylceramide (d18:1/16:0)	0.551	0.018	207.64	1.41	↑	ESI+
	11	Phosphatidylcholine (16:1(9Z)/16:0)	0.539	0.011	201.46	1.68	↑	ESI+
	12	Sphingomyelin (d18:1/18:1(9Z))	0.670	0.039	200.91	1.34	↑	ESI+
	13	d-xylose	1.943	0.025	154.16	2.00	↓	ESI−
	14	l-ribulose	2.090	0.009	315.92	2.52	↓	ESI−
FF	1	l-valine	2.965	0.000	302.69	1.92	↓	ESI−
	2	l-glutamine	0.449	0.034	406.31	1.47	↑	ESI+
	3	Ketoleucine	2.364	0.002	57.88	1.83	↓	ESI−
	4	Phenylpyruvic acid	1.618	0.033	92.35	1.41	↓	ESI−
	5	Gentisic acid	2.684	0.023	61.34	1.44	↓	ESI−
	6	Choline	0.568	0.010	280.86	1.70	↑	ESI+
	7	LysoPC (14:1(9Z))	0.379	0.0070	211.14	1.85	↑	ESI+
	8	Phosphatidylcholine (22:4(7Z,10Z,13Z,16Z)/14:0)	0.538	0.040	36.10	1.13	↑	ESI+
	9	PE (18:4 (6Z,9Z,12Z,15Z)/P-18:1(11Z))	0.303	0.015	58.74	1.68	↑	ESI+
	10	Arachidonic acid	0.309	0.044	46.47	1.45	↑	ESI−
	11	11,12-DiHETrE	1.824	0.010	78.28	1.66	↓	ESI−
	12	d-maltose	0.380	0.028	416.44	1.33	↑	ESI−
	13	6-Hydroxy-5-methoxyindole glucuronide	2.920	0.004	313.81	1.59	↓	ESI+
	14	Urocanic acid	0.468	0.020	322.52	1.73	↑	ESI+

IO: inactive ovaries; E: estrus; ID: identity; FC: fold change; RT: retention time; VIP: variable importance for the projection; FD: find the difference; ESI: electrospray ionization; FF: follicular fluid; PE: phosphatidyl ethanolamine; 11,12-DiHETrE: (5Z,8Z,14Z)-11,12-dihydroxyeicosa-5,8,14-trienoic acid. ^a^ is the VIP in the OPLS-DA model (VIP > 1); ^b^ is the *p*-value obtained by *t*-test (*p* < 0.05); ^c^ is the condition of serum metabolites in cows with IO, where “↓” indicates that the concentration of the IO group decreased; “↑” indicates that the concentration of the IO group increased.

## Data Availability

The raw data generated in this study can be obtained by reasonable request to the corresponding author.
